# Impact of food, alcohol and pH on modified-release hydrocortisone developed to treat congenital adrenal hyperplasia

**DOI:** 10.1530/EJE-16-0948

**Published:** 2017-01-17

**Authors:** Nayananjani Karunasena, Daniel N Margetson, Greg Neal, Martin J Whitaker, Richard JM Ross

**Affiliations:** 1 Department of Oncology and MetabolismUniversity of Sheffield, Sheffield, UK; 2 Diurnal LimitedCardiff, UK

## Abstract

**Background:**

We developed a modified-release hydrocortisone, Chronocort, to replace the cortisol rhythm in patients with congenital adrenal hyperplasia. Food, alcohol and pH affect drug absorption, and it is important to assess their impact when replicating a physiological rhythm.

**Subjects and methods:**

*In vitro* dissolution to study impact of alcohol and pH on Chronocort. A phase 1, three-period, cross over study in 18 volunteers to assess the impact of food on Chronocort and to compare bioavailability to immediate-release hydrocortisone.

**Results:**

*In vitro* dissolution of Chronocort was not affected by gastrointestinal pH up to 6.0 nor by an alcohol content up to 20% v/v. Food delayed and reduced the rate of absorption of Chronocort as reflected by a longer T_max_ (fed vs fasted: 6.75 h vs 4.5 h, *P* = 0005) and lower C_max_ (549.49 nmol/L vs 708.46 nmol/L, ratio 77% with CI 71–85). Cortisol exposure was similar in fed and fasted state: Geo LSmean ratio (CI) AUC_0t_ for fed/fasted was 108.33% (102.30–114.72%). Cortisol exposure was higher for Chronocort compared to immediate-release hydrocortisone: Geo LSmean ratios (CI) 118.83% (111.58–126.54%); however, derived free cortisol showed cortisol exposure CIs were within 80.0–125.0%: Geo LSmean ratio (CI) for AUC_0t_ 112.73% (105.33–120.65%).

**Conclusions:**

Gastric pH ≤6.0 and alcohol do not affect hydrocortisone release from Chronocort. Food delays Chronocort absorption, but cortisol exposure is similar in the fasted and fed state and exposure as assessed by free cortisol is similar between Chronocort and immediate-release hydrocortisone.

## Introduction

Cortisol has a distinct circadian rhythm with low levels at night, rising in the early hours of the morning, peaking on waking and declining over the day to low levels in the evening (1). Current immediate-release hydrocortisone therapy cannot replace this circadian rhythm; patients take twice or thrice daily hydrocortisone, and despite this, cortisol levels are suboptimal in many patients with adrenal insufficiency (2). This is a particular problem in patients with adrenal insufficiency due to congenital adrenal hyperplasia who have poor health outcomes (3). To address this, a number of technologies have been developed including hydrocortisone infusions (4), and modified-release formulations of hydrocortisone; Plenadren a once-daily modified-release hydrocortisone (5), and Chronocort a delayed and sustained absorption modified-release hydrocortisone that replicates the overnight profile of cortisol (6).

Food influences the absorption of immediate-release hydrocortisone; delaying and increasing the variability of the C_max_, prolonging the absorption half-life and decreasing oral clearance (7). Cortisone when taken with food shows an increased cortisol C_max_ and AUC; however, this was not considered to be clinically significant (8). In adrenal insufficiency, cortisol levels are low on waking at a time when they should be highest so most clinicians recommend taking hydrocortisone on waking although many patients will take food shortly afterward (7). The impact of food on modified-release formulations of hydrocortisone is likely to depend on the type of formulation developed. Plenadren is a dual release tablet with an immediate release coating and extended release core (5). Plenadren is recommended to be taken before breakfast and in the fasted state has approximately 20% lower bioavailability compared to immediate release hydrocortisone based on the AUC of cortisol (9). Plenadren taken with food shows similar changes to those seen with immediate-release hydrocortisone with a delayed C_max_ and an approximately 30% increase in AUC (10). Chronocort is a multi-particulate formulation that has a pH-dependent delayed release coating that allows for delayed release and sustained absorption (6). This manuscript reports the *in vitro* impact of alcohol and pH on the release of hydrocortisone from Chronocort, the *in vivo* impact of food on the pharmacokinetics of Chronocort and compares bioavailability to immediate-release hydrocortisone.

## Materials, methods and subjects

### *In vitro* dissolution studies of pH and alcohol

An *in vitro* dissolution methodology using rotating basket USP apparatus 1 (Sotax AT7 smart system) was developed to best represent the expected dissolution conditions *in vivo*. Briefly, dissolution vessels were filled with 700 mL of USP-simulated gastric fluid (pH 1.2) and pre-warmed to 37 ± 0.5°C. In separate vessels, sufficient buffer to adjust the pH of the media was pre-warmed. Chronocort (5 or 20 mg hydrocortisone) capsules were added to each basket and once the media was at the required temperature, they were lowered into the media, with the rotating speed set to 100 rpm. At 30-min intervals, 1–2 mL of sample was collected for analysis. The samples were vialled neat for analysis. The amount of hydrocortisone dissolved in the media was determined by HPLC analysis (Agilent 1100/1200 system) using reverse-phase chromatography with UV detection at 254 nm. The impact of gastric conditions and alcohol concentration on Chronocort was evaluated by dissolution testing under three different study conditions:

1. pH 1.2 for 0.5 h, pH-change to 4.5 for 2.5 h, pH-change to 7.2 for 1.5 h.
2. pH 4.5 for 3 h, pH-change to 7.2 for 1.5 h.
3. pH 1.2 for 2 h, pH-change to 6.0 for 1 h, pH-change to 7.2 for 1.5 h. All media were mixed with alcohol (ethanol) to produce a concentration of 5, 10, 20 or 40% v/v alcohol.

Calculation of dissolution profile comparison by *f*_2_ test was carried out according to the following formula:

where *R_t_* and *T_t_* are the cumulative percentage dissolved at each of the selected number (*n*) of time points of the reference dissolution profile and test dissolution profile respectively. Two dissolution profiles are considered similar when the similarity factor (*f*_2_) value is >50.

### *In vivo* pharmacokinetic studies fasted, fed and relative bioavailability

Human studies were undertaken at Simbec Research Ltd (Merthyr Tydfil, UK). Eighteen healthy male volunteers, mean age of 34 years (s.d. 9.3) and mean BMI of 24.91 kg/m^2^ (s.d. 1.8) participated in this open label, randomized, single-dose, three-period, cross over, single center, phase 1 study (www.clinicaltrials.gov trial Nbib2408068). The primary objectives of the study were to compare the pharmacokinetics of Chronocort under fed and fasted conditions at a single dose of 20 mg and to evaluate the relative bioavailability of Chronocort and immediate-release hydrocortisone. The three periods were Chronocort 20 mg in fed state (30 min after the start of a high-fat, high-calorie diet), Chronocort 20 mg in fasted state (10-h overnight fast) and hydrocortisone 20 mg in fasted state (10-h overnight fast). A minimal washout period of 7 days was ensured between each study period. Seventeen subjects completed the study, one subject was withdrawn due to treatment emergent adverse events (TEAE) after oral dexamethasone administration.

In study periods 1 and 2 (Chronocort 20 mg in fed state and fasted state) dexamethasone-suppressed subjects (1 mg dexamethasone administered at 22:00 h on Day 1, and at ~06:00, ~12:00, ~18:00 and ~22:00 h on Day 1) were admitted to the clinical unit on the previous day and remained until completion of all procedures (i.e. 24 h after dose). During the fed study subjects were required to fast for at least 10 h overnight and then receive a high-fat, high-calorie breakfast approximately 30 min before dosing in the morning of Day 1. During fasted study period, subjects were required to fast from 20:00 h on previous day until 4 h after dose on Day 1. Each dose of the study medication was administered at 08:00 h and 30 min after starting breakfast during the fed study period. A sample for basal blood serum cortisol was drawn pre dose (0 min–08:00 h) and further pharmacokinetic samples were drawn up to 24 h (every 30 min for 8 h, every 60 min up to 16 h and every 120 min up to 24 h).

In study period 3 (hydrocortisone fasted), dexamethasone-suppressed subjects (1 mg dexamethasone administered at 22:00 h on Day 1, and at ~06:00, ~12:00 and ~18:00 h on Day 1) were admitted to the clinical unit on the previous day and remained until completion of all procedures (i.e. 12 h after dose). Subjects fasted from 20:00 h on previous day until 4 h after dose on Day 1. Blood for serum cortisol were collected before dose (0 min–08:00 h) and up to 12 h (15 min, 30 min, 45 min, 1 h, 1.25 h, 1.5 h, 2 h, 2.5 h, 3 h, 4 h, 5 h, 6 h, 8 h, 10 h and 12 h).

### Assays

Cortisol was measured by LC-MS/MS using an Applied Biosystems MDS Sciex API365 mass spectrometer with Perkin Elmer Series 200 LC system with an electrospray source in negative ionization mode. Cortisol was measured in serum with the lower limit of quantitation (LLOQ) and upper limit of quantitation (ULOQ) of 0.50 and 250 ng/mL respectively. Reproducibility (relative standard deviation) RSD% was equal to <15% at the LLOQ QC (cortisol 0.50 ng/mL) and <10% at the low, medium and high QC level (cortisol 8, 30 and 200 ng/mL).

### Pharmacokinetics (PK)

It was calculated from serum cortisol and derived free cortisol by non-compartmental analysis, using baseline-adjusted concentrations. All calculations were done using WinNonlin Professional, version 5.3.

### Food effect and comparative bioavailability

After logarithmic transformation, C_max_, AUC_0−t_ and AUC_0−inf_ values were subjected to a mixed effects analysis of variance (ANOVA) including fixed effects for sequence, period and treatment and a random effect for subject nested within sequence. Point estimates and 90% confidence intervals (CI) were constructed for the contrasts between treatments using the residual mean square error obtained from the ANOVA. The point and interval estimates were then backtransformed to give estimates of the ratios of the geometric least squares means (LSmean) and corresponding 90% CI. In addition, estimated geometric means were produced for each treatment. An assessment of T_max_ was performed using the Wilcoxon matched-pairs test. In addition, a 95% non-parametric CI was constructed for the median difference in T_max_. Derived free cortisol was calculated for each sampling time point using the equation: y = 3E−20x^5 ^−1E−15x^4 ^+ 1E−11x^3 ^− 6E−08x^2 ^+ 0.0002x + 0.0306 (x = serum cortisol, y = derived free cortisol) (11). Serum cortisol and derived free cortisol were baseline adjusted using the 0-h sample for each subject, per treatment and values less than zero were set to zero. For calculation of derived PK parameters, concentrations below the lower limit of quantification were also assigned a value of zero.

### Ethics

All human studies were approved by the South East Wales Research Ethics Committee, and all participants gave written informed consent. The study protocol was authorised by the Medicines and Healthcare products Regulatory Agency (MHRA).

## Results

### *In vitro* impact of gastric pH

**Figure 1:**
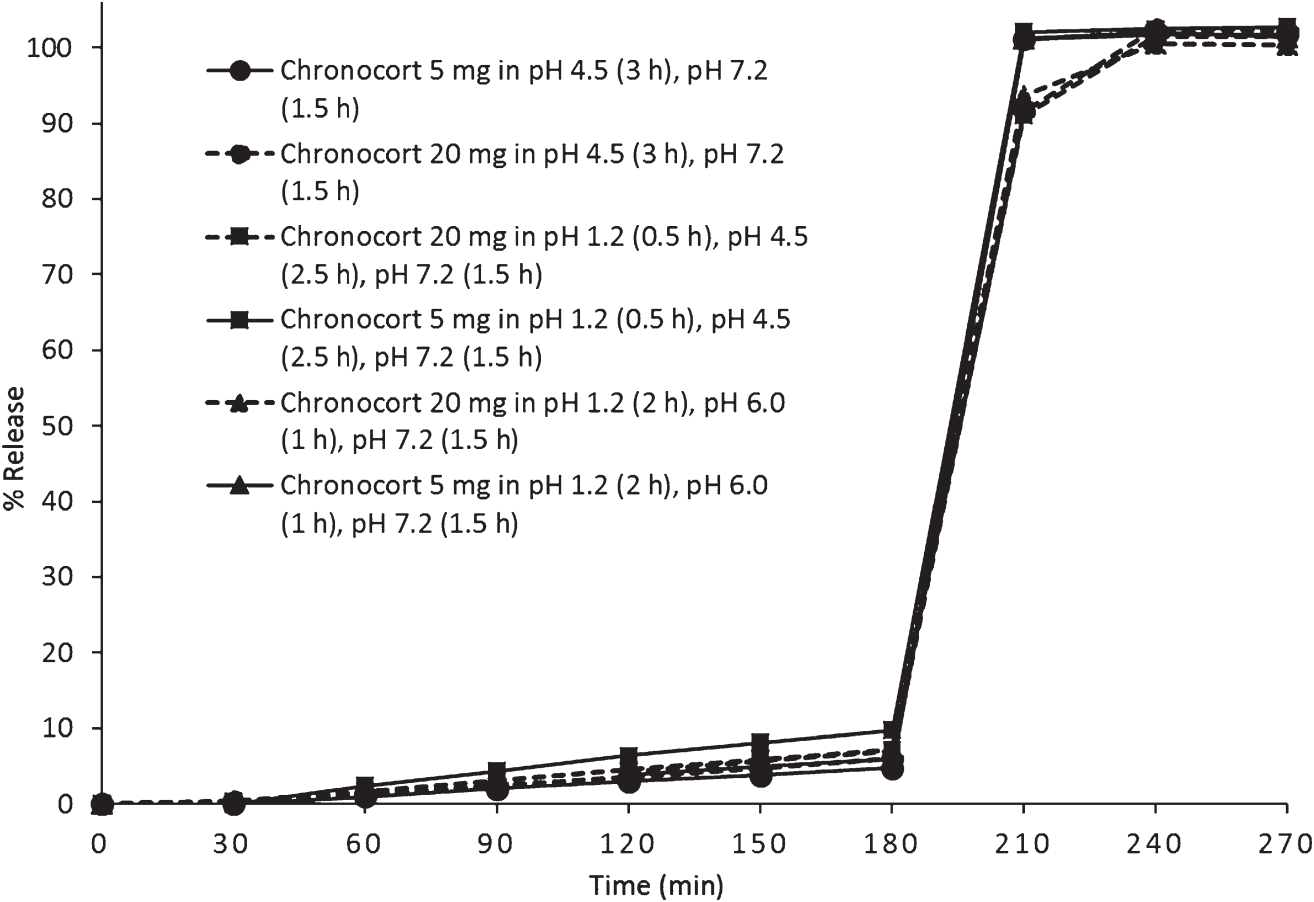
Chronocort 5 and 20 mg capsules *in vitro* dissolution (*n* = 6), mean hydrocortisone release in three different pH media conditions: (i) pH 1.2 (2 h), pH 6.0 (1 h), pH 7.2 (1.5 h); (ii) pH 1.2 (0.5 h), pH 4.5 (2.5 h), pH 7.2 (1.5 h); (iii) pH 4.5 (3 h), pH 7.2 (1.5 h). (s.d. error is within each point).

There was no significant drug release observed in pH 1.2 and 4.5 dissolution media prior to the third and final pH change to pH 7.2 media. The dissolution profiles of Chronocort 5 and 20 mg modified-release capsules were comparable across the range of physiological pH conditions studied. All dissolution profiles were comparable to the standard Chronocort dissolution profile (Fig. 1) when assessed using the *f*_2_ similarity test, with all values >50 (*f*_2_: 80–95). Chronocort 5 and 20 mg modified-release capsules continue to meet the compendial acceptance criteria for delayed-release dosage forms, demonstrating the integrity of the functional enteric coating was maintained in different acidic pH conditions and release of hydrocortisone was delayed until exposure to pH 7.2 media (Fig. 1).

### *In vitro* impact of alcohol

**Figure 2:**
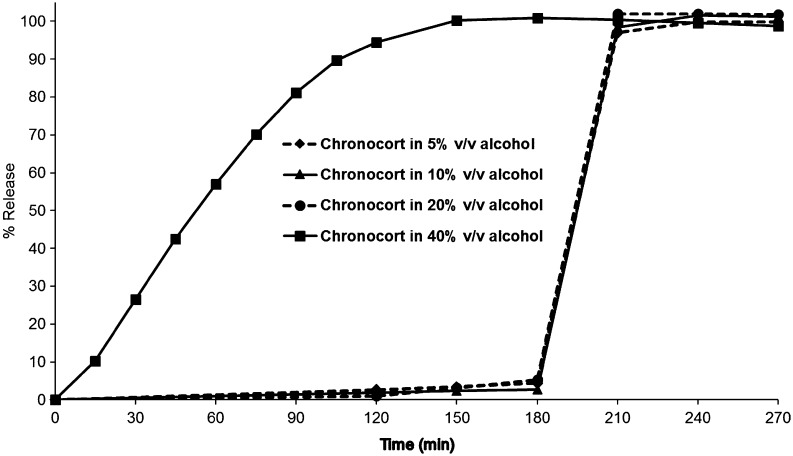
Chronocort 20 mg capsules *in vitro* dissolution in alcoholic media (*n* = 12), mean hydrocortisone release in 5, 10, 20 or 40% v/v alcohol mixed in pH 1.2 (2 h), pH 6.0 (1 h) and pH 7.2 (1.5 h) test conditions (s.d. error is within each point).

From 5 to 20% v/v of alcohol (ethanol) in the dissolution media, the obtained release profiles for Chronocort 20 mg modified-release capsules were all comparable to the standard Chronocort dissolution profile when assessed using the *f*_2_ similarity test, with all values >50 (*f*_2_: 68–70). The Chronocort modified-release formulation retains its gastro-resistant, enteric properties in up to 20% v/v alcohol over 3 h until media pH is changed to 7.2, triggering dissolution of the enteric coating and drug release (Fig. 2). A high concentration 40% v/v alcohol in the media led to a change in the dissolution profile, resulting in a value for the *f*_2_ similarity test outside of the acceptance criteria. However, only a moderate increase in drug release was observed over the dissolution experiment, with 27% drug release after 30 min, 57% after 1 h, 81% after 1.5 h and 95% after 2 h (*n* = 12 samples). At the high 40% v/v alcohol concentration, the rate of drug release increases, albeit without any observed dose dumping (Fig. 2).

### *In vivo* PK analysis

18 subjects who had concentrationtime profiles for 2 treatments and adequately suppressed pre-dose cortisol (<60 nmol/L) were included in the PK population. Hydrocortisone profiles of 4 subjects were excluded from the PK analysis of immediate-release hydrocortisone; two subjects did not suppress on one occasion each, one subject did not receive hydrocortisone tablets due to an adverse event after dexamethasone, one did not have a baseline sample performed.

### Food effect on Chronocort

**Figure 3:**
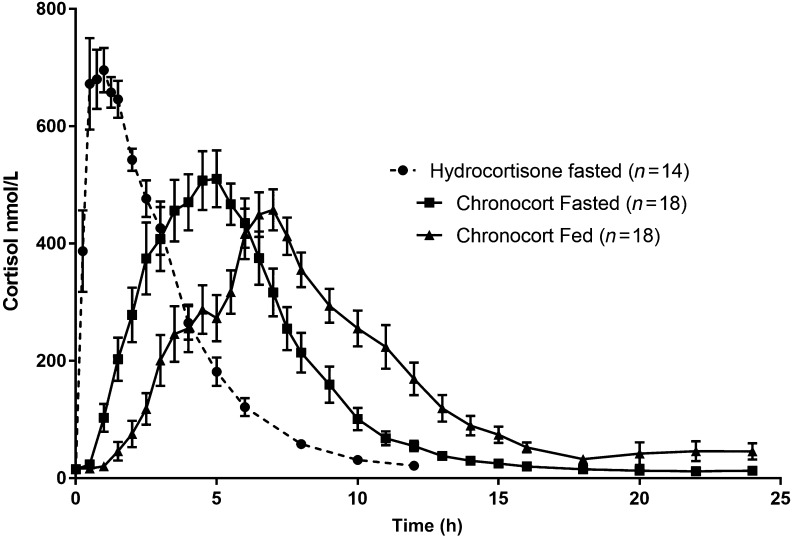
Mean (s.e.m.) cortisol concentration after immediate-release hydrocortisone fasted and Chronocort fasted and fed in healthy volunteers.

The rate of hydrocortisone absorption from Chronocort was reduced and delayed when administered in fed state (after a high-fat, high-calorie breakfast) when compared to the fasted state, as reflected by a lower C_max_ (549.49 nmol/L vs 708.46 nmol/L geometric LSmean ratio 77.56% with 90% C.I. 70.89–84.86%) and longer T_max_ (6.75 h vs 4.5 h, *P *= 0.0005) (Fig. 3 and Table 1). Geometric LSmean of AUC_0−t_ and AUC_0−inf_ were similar during fed (3229.26 and 2980.85 h*nmol/L) and fasted (3273.59 and 2985.31 h*nmol/L) study periods and the fed/fasted ratio CIs were within 80.0–125.0% indicating that the overall exposure to hydrocortisone was similar during fed and fasted periods. The t_1/2_ was the same for fed and fasted Chronocort administration at 1.38 h, and CL/F was similar at 16.70 and 18.48 L/h, respectively.

**Table 1:** Summary of the statistical analysis of food effect on Chronocort PK for serum cortisol (baseline adjusted) (PK population).

	IMPs: 20 mg hydrocortisone		
PK parameter	Chronocort (fed) (n = 18)*	Chronocort (fasted) (n = 18)*	Chronocort fed vs Chronocort fasted**
C_max_ (nmol/L)	549.49	708.46	77.56 (70.89–84.86)
AUC_0−t_ (h*nmol/L)	3229.26	2980.85	108.33 (102.30–114.72)
AUC_0−inf_ (h*nmol/L)	3273.59	2985.31	109.66 (103.19–116.53)
T_max_ (h)	6.75†	4.5†	2.25 (1.25 3.75) (0.0005)††

* Geometric LS mean; **Geometric LS mean (90%CI); ^†^Median; ^††^Median difference (95% CI) (*P* value).

### Comparative bioavailability of Chronocort vs hydrocortisone tablets—fasted

For serum cortisol, the geometric LSmean and test/reference ratio indicate that Chronocort is more bioavailable than the hydrocortisone tablets (Table 2). Overall exposure (AUC_0−t_ and AUC_0−inf_) is approximately 19% higher for Chronocort, as reflected by geometric LSmean ratios of approximately 119%. When compared to hydrocortisone tablets, the rate of absorption is also reduced and delayed after administration of Chronocort as reflected by a lower C_max_ (708.46 nmol/L vs 850.82 nmol/L, Geometric LSmean Ratio 74.75% with 90% CI of 64.08–87.19%) and longer T_max_ (4.5 h vs 0.88 h, *P *= 0.0014). The t_1/2_ was similar for Chronocort and hydrocortisone tablets at 1.38 and 1.40 h respectively. The CL/F appears to be higher for the hydrocortisone tablets when compared to Chronocort at 22.24 and 18.48 L/h respectively. Analysis of derived free cortisol suggests Chronocort exposure was within 80.0–125.0% CIs based on AUC_0−t_ and AUC_0−inf_ (Table 3).

**Table 2:** Summary of the statistical analysis of bioavailability between chronocort and hydrocortisone tablets (fasted) for serum cortisol (baseline adjusted) (PK population).

	IMPs: 20 mg hydrocortisone		
PK parameter	Chronocort (fasted) (n = 18)*	Hydrocortisone tablets (fasted) (n = 14)*	Chronocort fasted vs hydrocortisone tablets fasted**
C_max_ (nmol/L)	708.46	850.82	83.27 (75.58–91.74)
AUC_0−t_ (h*nmol/L)	2980.85	2508.52	118.83 (111.58–126.54)
AUC_0−inf_ (h*nmol/L)	2985.31	2510.82	118.90 (111.58–126.69)
T_max_ (h)	4.5†	0.88†	3.50 (2.25–4.38) (0.0014)††

*Geometric LS mean; **Geometric LS mean (90%CI); ^†^Median; ^††^Median difference (95% CI) (*P* value).

**Table 3:** Summary of the statistical analysis of bioavailability between chronocort and hydrocortisone tablets (fasted) for derived free cortisol (baseline adjusted) (PK population).

	IMPs: 20 mg hydrocortisone		
PK parameter	Chronocort (fasted) (n = 18)*	Hydrocortisone tablets (fasted) (n = 14)*	Chronocort fasted vs hydrocortisone tablets fasted**
C_max_ (nmol/L)	106.06	141.89	74.75 (64.08–87.19)
AUC_0−t_ (h*nmol/L)	326.87	289.96	112.73 (105.33–120.65)
AUC_0−inf_ (h*nmol/L)	327.03	290.36	112.63 (105.39–120.36)
T_max_ (h)	4.5†	0.88†	3.50 (2.25–4.38) (0.0014)††

*Geometric LS mean; **Geometric LS mean (90%CI); ^†^Median; ^††^Median difference (95% CI) (*P* value).

## Discussion

Chronocort modified-release *in vitro* dissolution was neither affected by prolonged exposure to gastric dissolution media up to pH 6.0 nor by an alcohol content up to 20% v/v, demonstrating that the polymer system used to provide the delayed release functionality affords a robust enteric performance without resulting in premature coat dissolution or affecting the downstream release of hydrocortisone. Taking Chronocort with food delayed and reduced the cortisol C_max_ but did not affect the overall cortisol exposure. Chronocort showed greater comparative bioavailability when compared to immediate-release hydrocortisone although this was within 80–125% CIs when adjusted for protein binding.

Gastric pH in healthy individuals may vary between pH 1.0 and 2.5 preprandial and then increases as food is ingested to reach a pH of ≤5.0 before returning to normal gastric pH values; after gastric emptying, medication passes through the small intestine where pH values in the distal ileum are reported to vary between pH 6.5 and 8.0 (12, 13). Chronocort was designed to release hydrocortisone in the last third of the small bowel to allow for delayed and sustained absorption (6). The multi-particulate formulation approach was selected to provide more predictable, reproducible gastrointestinal transit over single-unit oral dosage forms (14). To achieve its release profile, the multi-particulate beads of Chronocort are coated with the polymer EUDRAGIT (S100/L100) designed to dissolve at pH ≥6.8. Our data presented here demonstrate that the enteric coat does not dissolve at pH values ≤6.0 and drug release only occurs above the enteric coat pH 6.8 trigger point. This is of importance suggesting that there should not be a problem taking Chronocort with concomitant medication that raises gastric pH, such as proton pump inhibitors where the gastric pH can typically rise above 4.0 but not > than pH 6.0 (15, 16).

Alcohol can influence drug absorption from modified-release dosage forms and potentially on the dissolution of the enteric coating of drugs (17). Concomitant consumption of alcoholic beverages with such products may induce dose dumping (18). We examined the effect of alcohol on the dissolution of Chronocort as it is designed to be taken last thing at night to provide the overnight rise in cortisol, and in many cultures, it is common to have alcohol in the evening. From 5 to 20% v/v of alcohol, there was no effect of alcohol on hydrocortisone release confirming that Chronocort meets the EMA regulatory guidelines for alcohol testing of modified-release dosage forms (18). At a high alcohol concentration of 40% v/v recommended in the FDA guideline for testing (19), there was a change in the Chronocort dissolution profile observed, with a moderate increase in drug release over the 2 h but no significant alcohol-induced dose dumping. The conclusion from this study, is that dissolution of Chronocort modified-release capsules are not perturbed by alcohol under typical consumption levels and shows no dose dumping. The strongest alcoholic spirits generally available for consumption are ca. 40% v/v alcohol which on ingestion would be very rapidly diluted to <20% v/v in the stomach. It has been calculated that to achieve a 40% v/v alcohol concentration in the stomach would require the intake into an empty stomach of 240 mL of an alcoholic beverage with 56% v/v alcohol content, based on 100 mL gastric fluid (20). Furthermore, hydrocortisone has a wide therapeutic index and long established safety profile, lowering the risk profile for Chronocort modified-release capsules. For example, for some indications, i.e. asthma and COPD, doses up to 500 mg are used. Therefore, the concomitant consumption of alcoholic beverages presents no risk for Chronocort dose dumping.

Food is known to slow the absorption of many drugs as it slows gastric emptying and gut transit (14). We found that a high-fat, high-calorie meal delayed absorption of Chronocort but did not alter the overall cortisol exposure. For immediate-release hydrocortisone, the evidence shows that food delays the C_max_ and increases exposure (7), and the same thing was true for the dual-release formulation of hydrocortisone Plenadren (10). The median time of T_max_ for Chronocort given with food was 6.75 h and when fasted was 4.5 h. We have previously published data that shows when Chronocort 20 mg was given at 23:00 h at night a median T_max_ of 6 h was achieved, similar to that when Chronocort 20 mg was taken in the morning with food. It is likely that a night-time dose has a delayed T_max_ despite being without food due to increased gastric residence and slower gut motility during sleep (21). It is likely in clinical practice that patients will take Chronocort in the morning close to food, and this may give a delayed T_max_ but will not increase exposure to cortisol.

Comparative bioavailability showed that Chronocort taken in fasted state gave greater cortisol exposure than immediate-release hydrocortisone, but when adjusted for protein binding, they gave similar cortisol exposure. This would be predicted as serum cortisol is highly protein bound and binding to plasma proteins is non-linear with higher plasma concentrations having a greater unbound fraction than lower concentrations (11), and the unbound fraction having more rapid clearance (22). When taking 20 mg immediate-release hydrocortisone, the C_max_ exceeds the binding capacity of cortisol-binding protein at a concentration of ~550 nmol/L, the free cortisol is more quickly cleared than the bound fraction and therefore the cortisol AUC for total cortisol is lower than it would be if it had not exceeded the binding capacity (11). In contrast, Chronocort gives a C_max_ that is comparable to healthy subjects, and there is not such a great free portion although with its sustained absorption, the free cortisol exposure over time is similar to immediate release hydrocortisone. Thus, Chronocort, designed with a robust enteric coat, is unaffected by normal physiological pH and alcohol conditions encountered in normal daily life whilst providing cortisol replacement to levels which are similar to those found in healthy individuals.

## Declaration of interest

R J R and M J W are directors and D N M and G N are employees of Diurnal Ltd.
